# Single‐Atom Control of Aromaticity and Excited‐State Dynamics in Endohedral Zirconium–Antimony Zintl Clusters

**DOI:** 10.1002/anie.6088506

**Published:** 2026-08-06

**Authors:** Yun Zhang, Ziqi Deng, Wen‐Juan Tian, Hui‐Qing Sun, Kang Sun, Alvaro Muñoz‐Castro, Zhong‐Ming Sun, Teng‐Teng Chen

**Affiliations:** ^1^ State Key Laboratory and Institute of Elemento‐Organic Chemistry, Tianjin Key Lab of Rare Earth Materials and Applications School of Material Science and Engineering Nankai University Tianjin China; ^2^ Department of Chemistry The Hong Kong University of Science and Technology Hong Kong China; ^3^ Institute of Molecular Science Shanxi University Taiyuan China; ^4^ Facultad De Ingeniería Universidad San Sebastián Santiago Chile; ^5^ Department of Chemistry Fudan University Shanghai China; ^6^ IAS Center for Quantum Matter The Hong Kong University of Science and Technology Hong Kong China

**Keywords:** antimony, femtosecond transient absorption, spherical aromaticity, superatomic clusters, zintl clusters

## Abstract

Three‐dimensional (3D) aromaticity provides a powerful framework for understanding the stability and electronic structures of superatomic clusters, yet how such aromaticity evolves during atom‐by‐atom structural growth remains poorly understood. Herein, we report two endohedral zirconium‐antimony Zintl clusters, [Zr@Sb_12_]^2−^ and [Zr@Sb_13_]^3−^, that offer a rare one‐atom comparison of nuclearity‐dependent aromaticity and photodynamics. Structural and bonding analyses reveal that [Zr@Sb_12_]^2−^ behaves as an integrated Sb_12_ framework rather than three discrete Sb_4_ fragments coordinated to Zr. Adaptive natural density partitioning (AdNDP) and magnetic‐response analyses establish a closed‐shell S^2^P^6^ superatomic configuration, giving rise to pronounced spherical all‐metal aromaticity. In contrast, incorporation of one additional Sb atom reorganizes the framework into a cage‐like [Zr@Sb_13_]^3−^ cluster, disrupts superatomic shell closure, and produces an S^2^P^4^ configuration with strongly attenuated spherical aromaticity. Femtosecond transient absorption (fs‐TA) spectroscopy further reveals that the aromatic [Zr@Sb_12_]^2−^ cluster exhibits markedly longer‐lived excited‐state species than [Zr@Sb_13_]^3−^. These findings establish a direct structure–aromaticity–dynamics relationship, demonstrating that single‐atom control of cluster nuclearity can regulate not only ground‐state aromatic stabilization but also excited‐state robustness in all‐metal superatoms.

## Graphical abstract

Single‐atom growth from [Zr@Sb_12_]^2−^ to [Zr@Sb_13_]^3−^ disrupts the closed‐shell S^2^P^6^ superatomic configuration and markedly attenuates three‐dimensional (3D) aromaticity. Ultrafast spectroscopy further links stronger spherical aromaticity to slower excited‐state relaxation, establishing an atom‐resolved structure‐aromaticity‐dynamics relationship.

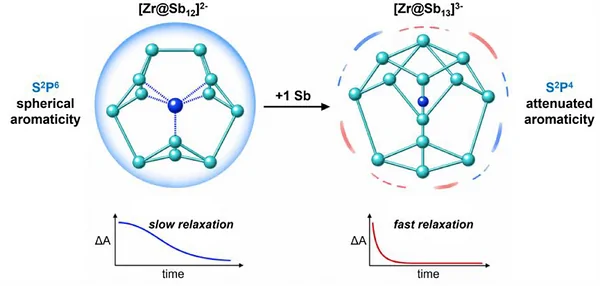

## 1 Introduction

Atomically precise nanoclusters often described as “superatoms”, provide a molecular‐level platform for bridging the gap between discrete molecules and extended solids. In contrast to conventional nanoparticles, whose structures and properties are often obscured by size and compositional distributions, superatomic clusters possess well‐defined nuclearity, composition, and electronic structure. Their valence electrons occupy discrete shell‐like orbitals that resemble atomic s‐, p‐, d‐, and f‐orbitals. The filling of these shell‐like orbitals with “magic numbers” of electrons (e.g., 2, 8, 18, 32) results in closed‐shell configurations, leading to enhanced thermodynamic stability, enlarged enengy gap between the highest occupied molecular orbital (HOMO) and the lowest unoccupied molecular orbital (LUMO), reduced reactivity, and distinctive optical or catalytic properties [1]. Because the addition or removal of even a single atom can reorganize the electronic shell structure, superatomic clusters provide an ideal platform for understanding how atomic‐level structural changes control collective electronic behavior.

Aromaticity is one of the most powerful concepts for describing electron delocalization and stability in chemistry. Although originally developed for planar organic molecules [2, 3, 4, 5], this concept has been extended far beyond benzene‐like π systems to include boranes, [6, 7] carboranes, [8, 9] fullerenes, [10, 11], Zintl ions [12], and all‐metal clusters. In particular, three‐dimensional (3D) aromaticity has emerged as a key framework for understanding highly delocalized cage‐like systems. For spherical aromatic species, the 2(*N* + 1)^2^ Hirsch rule predicts closed‐shell stabilization at electron counts of 2, 8, 18, 32, and 50 [13]. In such systems, aromaticity is reflected not only in geometric and electronic structure, but also in magnetic responses, including characteristic interior shielding, negative nucleus‐independent chemical shift values, and diatropic current‐density patterns [14, 15, 16, 17]. More broadly, 3D electronic delocalization can generate shielding responses that persist under different orientations of an external magnetic field [13], extending the classical concept of the shielding cone from planar aromatic hydrocarbons to nonclassical molecular clusters [18, 19, 20, 21, 22, 23, 24, 25, 26]. Thus, spherical aromaticity provides a conceptual bridge between organic and inorganic chemistry and offers a powerful framework for understanding the stability of all‐metal superatomic clusters.

Despite these advances, a fundamental question remains: how robust is 3D aromaticity during atom‐by‐atom structural evolution? [27, 28, 29] Most known spherical aromatic clusters have been examined as isolated end points, whereas the structural and electronic consequences of changing cluster nuclearity by a single atom remain difficult to capture experimentally. Because 3D aromaticity depends sensitively on electron count, orbital degeneracy, and symmetry, even a subtle change in nuclearity may disrupt superatomic shell closure and substantially alter aromatic stabilization. Directly comparison of two closely related clusters that differ by only one framework atom would therefore provide a rare opportunity to determine how aromaticity emerges, persists, or collapses during cluster growth. Zintl clusters are particularly attractive for addressing this question. These soluble main‐group polyanionic clusters exhibit rich structural diversity, flexible electron counts, and extensive multicenter bonding. Their solution synthesis, often involving the dissolution or transformation of Zintl‐phase precursors in polar solvents in the presence of sequestering agents such as [2.2.2]‐crypt (4,7,13,16,21,24‐hexaoxa‐1,10‐diazabicyclo[8.8.8]hexacosane), enables the isolation of well‐defined anionic clusters as molecular building blocks. Introducing transition‐metal precursors into Zintl chemistry offers additional opportunities to stabilize endohedral intermetalloid clusters with unusual geometries and electronic structures. These features make Zintl clusters promising templates for probing atom‐by‐atom growth, all‐metal aromaticity, and superatomic structure–property relationships in the condensed phase.

Herein, we report the synthesis, structural characterization, electronic analysis, and ultrafast spectroscopic investigation of two endohedral zirconium–antimony Zintl clusters, [K([2.2.2]‐crypt)]_2_[Zr@Sb_12_] (**1**) and [K([2.2.2]‐crypt)]_3_[Zr@Sb_13_] (**2**). These clusters provide an atom‐resolved snapshot of structural evolution from a [Zr@Sb_12_]^2−^ framework to a [Zr@Sb_13_]^3−^ cage. Electronic‐structure and magnetic‐response analyses show that [Zr@Sb_12_]^2−^ possesses a closed‐shell S^2^P^6^ superatomic configuration and exhibits pronounced spherical all‐metal aromaticity. In contrast, incorporation of one additional Sb atom reorganizes the cluster framework and disrupts superatomic shell closure, giving [Zr@Sb_13_]^3−^ an S^2^P^4^ configuration with attenuated delocalization and nonspherical aromatic character [16, 30]. Femtosecond transient absorption (Fs‐TA) spectroscopy further reveals that the aromatic [Zr@Sb_12_]^2−^ cluster undergoes substantially slower excited‐state relaxation than its [Zr@Sb_13_]^3−^ counterpart. These results establish a direct connection between single‐atom structural evolution, 3D aromaticity, and excited‐state dynamics, showing that aromaticity can influence not only ground‐state stability but also the photophysical robustness of all‐metal superatoms.

## 2 Results and Discussion

Compound **1**, [K([2.2.2]‐crypt)]_2_[Zr@Sb_12_], was synthesized by reacting K_5_Sb_4_ with ZrCp_2_Cl_2_ in ethylenediamine (en) in the presence of [2.2.2]‐crypt (Figure 1). Stirring the reaction mixture at room temperature for 5 h, followed by crystallization over three weeks, afforded black needle‐shaped crystals in 13% yield. Compound **2**, [K([2.2.2]‐crypt)]_3_[Zr@Sb_13_] was obtained under otherwise similar conditions, except that the reaction was conducted at 60°C for 3 h. Subsequent crystallization over three weeks yielded black block‐shaped crystals in 26% yield. To further investigate the possible structural evolution relationship between these two clusters, the reaction system of compound **1** was subjected to additional thermal treatment by introducing ectra K_5_Sb_4_ and heating at 60°C under continuous stirring for an additional 1 h. Notably, trace amounts of the [Zr@Sb_13_]^3^^−^ species were identified during this process, accompanied by the formation of a substantial amount of black amorphous precipitate. These observations suggest that [Zr@Sb_12_]^2^^−^ may undergo thermally induced cluster reconstruction and atom‐by‐atom growth under elevated‐temperature conditions. Suitable single crystals of **1** and **2** were analyzed by single‐crystal x‐ray diffraction, which enabled unambiguous structural determination. Energy‐dispersive x‐ray spectroscopy (EDX, Figure S6) further supports the elemental compositions derived from the crystallographic analysis. Attempts to confirm the cluster compositions by electrospray ionization mass spectrometry (ESI‐MS) were unsuccessful, as no signals assignable to the intact parent anions were detected, most likely because of dissociation under the ionization conditions.

**FIGURE 1:**
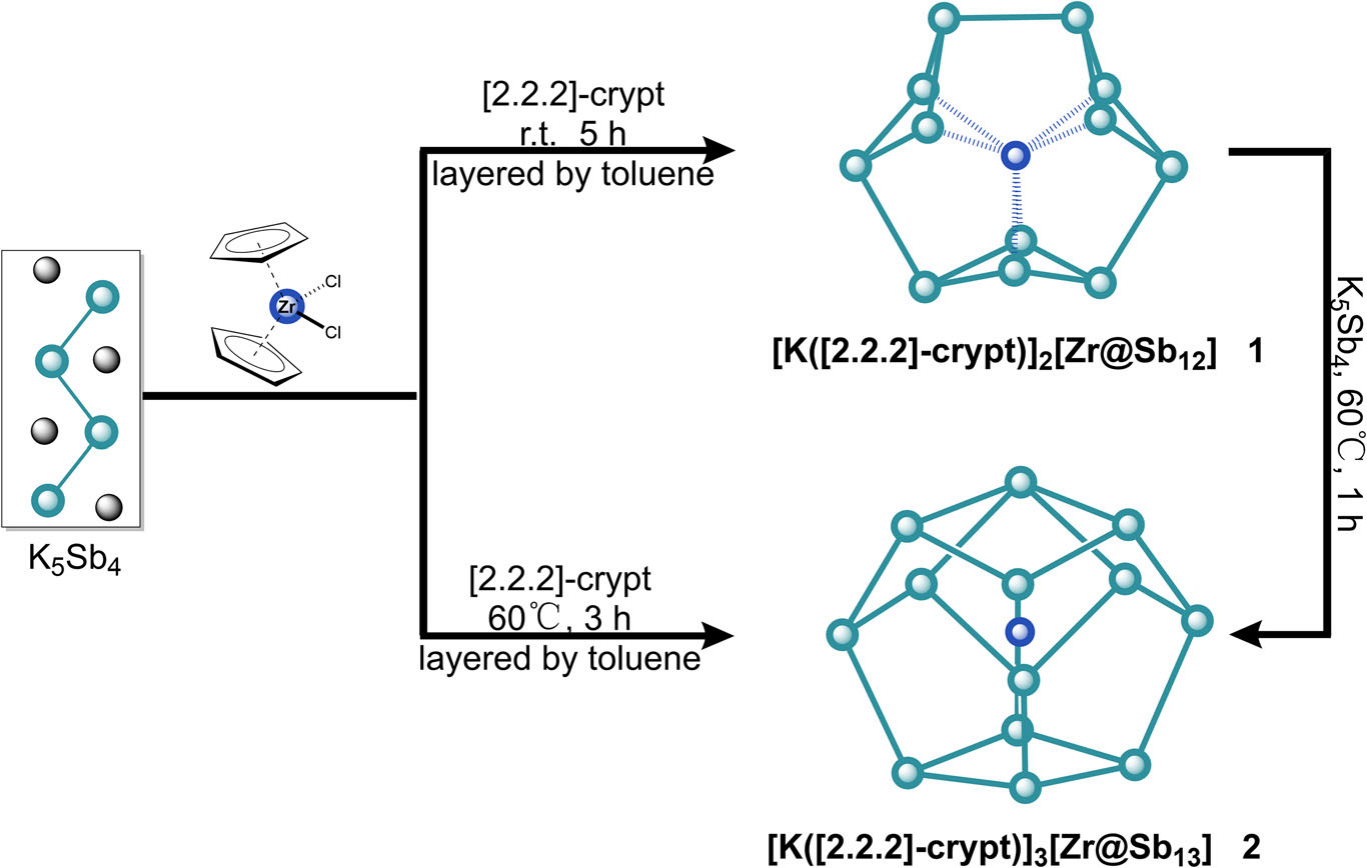
Synthetic preparation of the [K([2.2.2]‐crypt)]_2_Zr@Sb_12_ (**1**) and [K([2.2.2]‐crypt)]_3_Zr@Sb_13_ (**2**).

Single‐crystal x‐ray diffraction reveals that compound **1** crystallizes in the triclinic space group *P* $\bar{1}$. The asymmetric unit contains two K([2.2.2]‐crypt)^+^ cations, one [Zr@Sb_12_]^2−^ cluster anion, and one toluene molecule. The [Zr@Sb_12_]^2−^ anion is structurally related to previously reported endohedral tetrel/pnictogen clusters, including [Ln(*η* ^4^‐Sb_4_)_3_]^3−^ (Ln = La, Y, Ho, Er, Lu) [31] and [An@Bi_12_]^3−/4−^ (An = U, Th) [32, 33]. Structurally, the cluster consists of three fused and folded Sb_4_ units that assemble into an Sb_12_ framework encapsulating a central Zr atom (Figure 2a). In the reported [Ln(*η* ^4^‐Sb_4_)_3_]^3−^ clusters, the Sb_12_ framework contains two crystallographically and chemically distinct sets of Sb atoms, commonly described as six equatorial and six latitudinal sites. The Sb–Sb bond lengths within the Sb_4_ units, 2.8088(5)–2.8339(6) Å are significantly shorter than the contacts between adjacent Sb_4_ units, namely those between equatorial Sb atoms, 3.0179(6)–3.0517(5) Å. A related but more delocalized bonding pattern is observed in [Zr@Sb_12_]^2−^. The intra‐ring Sb–Sb distances, 2.8028(4)–2.8381(3) Å are comparable to those in [Ln(*η* ^4^‐Sb_4_)_3_]^3−^, whereas the inter‐ring Sb–Sb separations are contracted to 2.9502(4)–2.9552(4) Å. This contraction suggests enhanced bonding interactions between adjacent Sb_4_ units, likely involving π‐type delocalization across the Sb_12_ framework. Similar bond‐length equalization has been observed in aromatic [Th@Bi_12_]^4−^ clusters, indicating that the stability of cluster **1** may, at least in part, arise from aromatic stabilization within the pnictogen cage.

**FIGURE 2:**
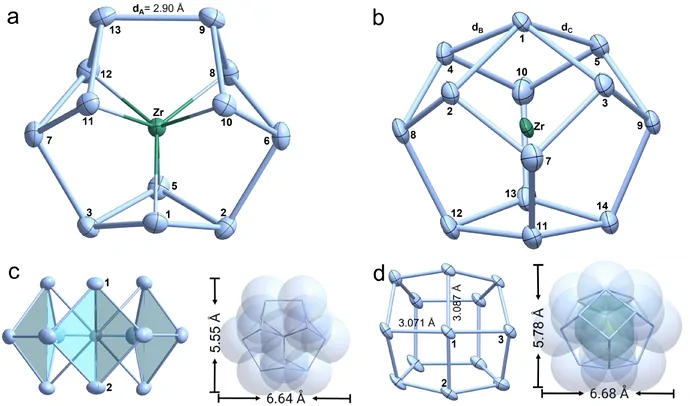
Molecular structures of [Zr@Sb_12_]^2−^ and [Zr@Sb_13_]^3−^. (a, b) Top view of [Zr@Sb_12_]^2−^ and [Zr@Sb_13_]^3−^, respectively; thermal ellipsoids are drawn at the 50% probability level. (c, d) Alternative views and space‐filling representations of [Zr@Sb_12_]^2−^ and [Zr@Sb_13_]^3−^, respectively; the labeled values denote the van der Waals dimensions along the indicated directions.

Compound **2** crystallizes in the monoclinic space group *P*2_1_/m. The asymmetric unit comprises one [Zr@Sb_13_]^3−^ cluster anion, three K([2.2.2]‐crypt)^+^ cations, and one toluene molecule. The [Zr@Sb_13_]^3−^ anion adopts a closed cage structure, with the Zr atom located near the center of the Sb_13_ framework. Structurally, the cage can be described as four fused Sb_5_ units capped by an additional Sb atom (Figure 2b). The Sb–Sb distances within the Sb_5_ units range from 2.7974(10) to 2.8714(9) Å, whereas the distances between the apical Sb atom and adjacent Sb_5_ units are 3.078(1) and 3.0871(9) Å for *d*_B_ and *d*_C_, respectively (Figure 2d). The isolation of such a high‐nuclearity all‐antimony cage in the condensed phase is notable. In analogy to the theoretically predicted P_14_ cage [34], the construction of 3D group 15 cages is challenging because pnictogen atoms tend to favor locally planar or pyramidal coordination environments, while the formation of expanded five‐membered‐ring motifs imposes considerable angular strain, with bond angles approaching approximately 108°. Although a limited number of Zintl clusters with similar endohedral lanthanide motifs have been reported, these systems generally rely on heteroatom incorporation to tune the electron count and stabilize the cluster framework. For example, the formation of [Sm@Ga_3‐x_H_3‐2x_Bi_10+x_]^3−^ (*x* = 0, 1) depends on Ga/Bi Lewis interactions to mitigate excessive negative charge density at Ga^2−^ sites [35]. By contrast, [Zr@Sb_13_]^3−^ represents the first all‐antimony cage composed exclusively of Group 15 elements. No additional main‐group heteroatoms is required to tune the electron count for the cage. Instead, the encapsulated Zr center participates directly in multicenter Zr‐Sb bonding, and the resulting mixing of Zr 4d and Sb 5p orbitals helps stabilize the closed Sb_13_ framework.

From a structural‐evolution perspective, [Zr@Sb_13_]^3−^ can be regarded as an expanded derivative of [Zr@Sb_12_]^2−^. The transformation can be visualized as fusion of additional Sb‐based fragments through shared Sb vertices, accompanied by incorporation of two Sb atoms that serve as top and bottom caps, ultimately yielding a closed 3D cage structure (Figure S9). This relationship highlights not only precise nuclearity control during atom‐by‐atom assembly, but also the coupled evolution of geometry, electron count, and framework stabilization in endohedral Zintl clusters.

The experimentally characterized [Zr@Sb_12_]^2−^ cluster can be described as a central Zr atom embedded within a distorted Sb_12_ rim of approximate *D*_3h_ symmetry. The structure contains two sets of Zr–Sb distances, 2.971 and 3.333 Å, and two sets of Sb–Sb distances, 2.824 and 2.949 Å. Density functional calculations indicate that this structure is strongly favored over a hypothetical spherical icosahedral structure of *I*_h_ symmetry by 146.8 kcal/mol at the PBE0/STO‐TZ2P level. This energetic preference is mainly attributed to the substantially weakened orbital bonding interaction in the icosahedral alternative (Table S4). The three Sb_4_ units in [Zr@Sb_12_]^2−^ deviate markedly from planarity, forming a bowl‐shaped geometry with a depth of 0.614 Å (Figure S8). This distortion is significantly larger than that observed in the related [La(*η* ^4^‐Sb_4_)_3_]^3−^ cluster, [31] in which the Sb_4_ units show a smaller bowl depth of 0.332 Å. In [La(*η* ^4^‐Sb_4_)_3_]^3−^, the structure can be viewed as a central metal ion coordinated by three relatively discrete Sb_4_ rings. In contrast, the pronounced distortion in [Zr@Sb_12_]^2−^ suggests stronger integration of the Sb_4_ fragments into a single delocalized Sb_12_ framework rather than a simple coordination assembly of three independent Sb_4_ ligands around central Zr metal center. Consistently, electron density of delocalized bonds (EDDB) analysis assigns 14.9 |*e*| to global delocalization in the cluster (EDDB_G_), of which 12.8 |*e*| are associated primarily with the Sb_12_ rim fragment (EDDB_F_; Figure S12). This agreement with the magnetic‐response analysis (*vide infra*) further supports a delocalization pattern concentrated predominantly within the Sb_12_ framework.

To elucidate the bonding patterns in [Zr@Sb_12_]^2−^, adaptive natural density partitioning (AdNDP) analysis was performed (Figure 3). The [Zr@Sb_12_]^2−^ cluster contains a total of 66 valence electrons, calculated as 4 electrons from Zr, 60 electrons from 12 Sb atoms, and 2 additional electrons from the overall charge. These electrons are first assigned to twelve Sb 5s lone pairs, represented as 1c–2e bonds, and 12 localized Sb–Sb bonds, represented as 2c–2e elements within the Sb_12_ cluster rim. This bonding description is partially analogous to that proposed for [La(*η* ^4^‐Sb_4_)_3_]^3−^ [31]. In addition to these localized bonds, AdNDP identifies five 7c–2e delocalized bonds involving the central Zr atom and the six closest Sb atoms. These multicenter bonds arise from mixing between radial Sb 5p and Zr 4d orbitals and provide substantial Zr‐Sb_12_ metal‐framework stabilization. Their presence is consistent with the enhanced distortion of the Sb_4_ units and their stronger integration into the Sb_12_ framework relative to the weaklier coupled Sb_4_ fragments in the [La(*η* ^4^‐Sb_4_)_3_]^3−^ cluster [31]. The remaining eight electrons are accommodated in four 12c–2e delocalized bonding elements distributed over the Sb_12_ framework. These bonds involve tangential Sb 5p orbitals and extend across the entire Sb_12_ rim, supporting the description of [Zr@Sb_12_]^2−^ as an integrated cluster rather than a collection of three electronically isolated Sb_4_ rings as in [La(*η* ^4^‐Sb_4_)_3_]^3−^ [31]. The four 12c–2e delocalized bonds contain eight delocalized electrons, satisfying the 2(*N*+1)^2^ Hirsch rule for 3D aromaticity with *N* = 1 [22]. Accordingly, [Zr@Sb_12_]^2−^ can be described as a spherically aromatic cluster. Its delocalized electronic structure is analogous to the shell structure of isolated atoms, comprising one totally symmetric orbital with zero angular nodes and three orbitals with one angular node. This corresponds to a filled S^2^P^6^ superatomic shell, identifying [Zr@Sb_12_]^2−^ as a superatomic aromatic cluster.

**FIGURE 3:**
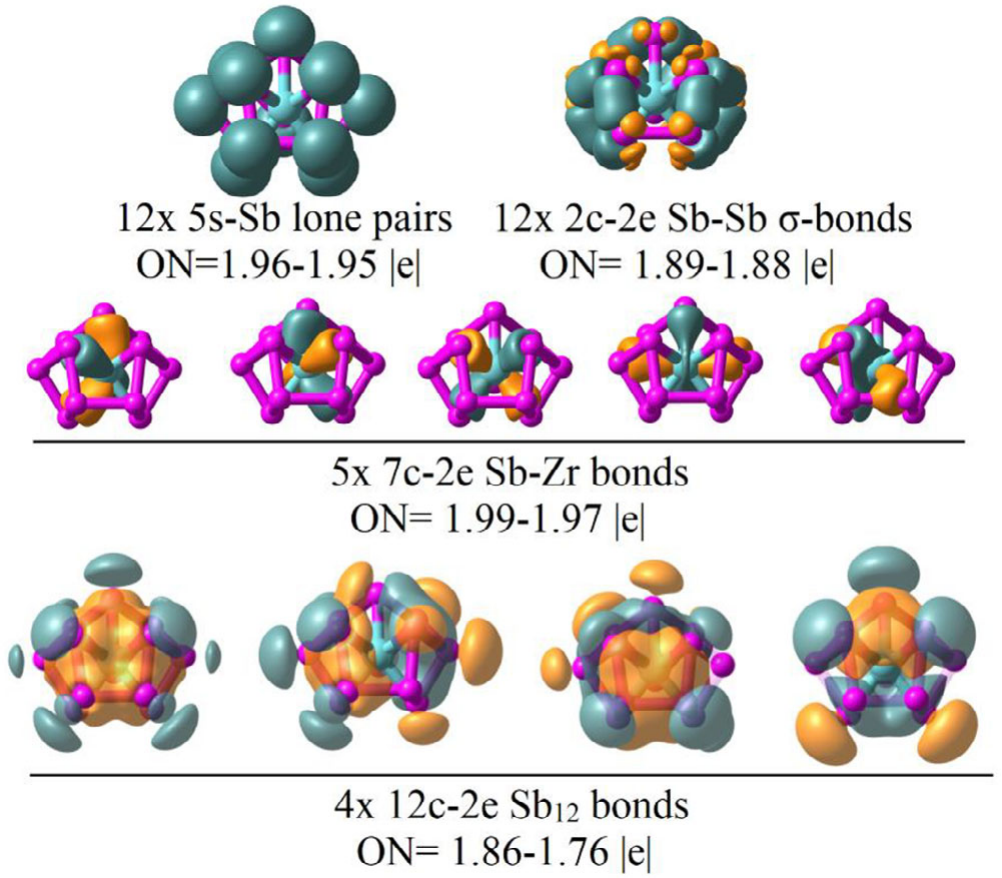
Bonding elements from AdNDP calculations for the [Zr@Sb_12_]^2−^ cluster. ON stands for occupation numbers, given in electrons.

To further evaluate the aromatic character associated with the delocalized bonding in [Zr@Sb_12_]^2−^, the induced magnetic response was analyzed (Figure 4). The spatial distribution of the induced magnetic field provides a sensitive probe of aromaticity, because aromatic systems generally generate a shielding region within the molecular or cluster interior [36, 37, 38, 39, 40, 41]. The isotropic component of the induced magnetic field **B** ^ind^ (**B**_iso_^ind^), commonly expressed as the nucleus‐independent chemical shift (NICS) [42], reflects the orientationally averaged magnetic response relevant to molecules undergoing rapid tumbling in solution. For [Zr@Sb_12_]^2−^, the NICS isosurface shows a continuous shielding region confined within the overall Sb_12_ framework. This feature is characteristic of spherical aromatic species [40] and is consistent with the favorable delocalized bonding pattern expected for an S^2^P^6^ superatomic configuration [43].

**FIGURE 4:**
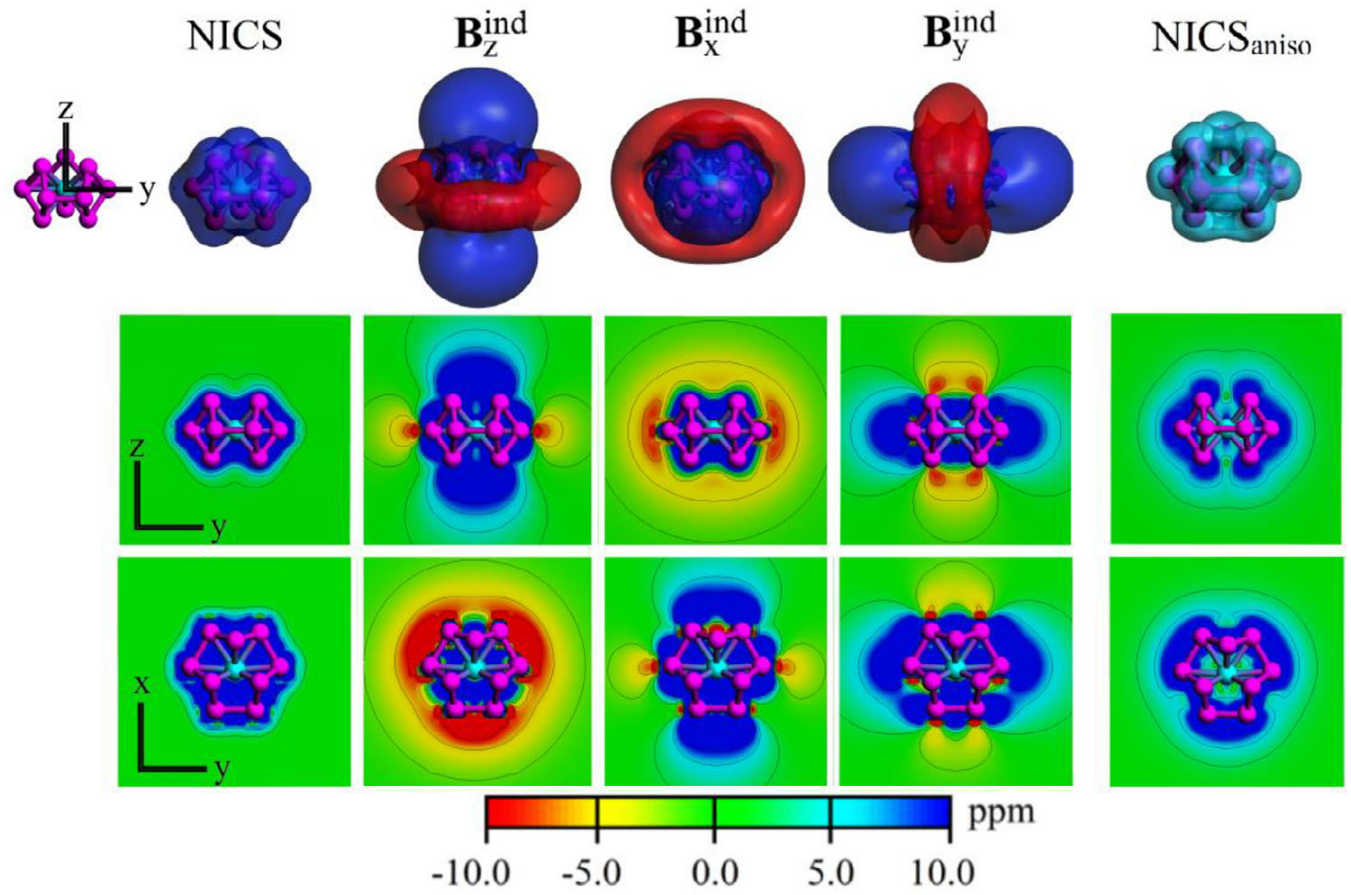
Magnetic response properties for [Zr@Sb_12_]^2−^, accounting for NICS, and under certain representative orientations of the external field (B_x_^ind^, B_y_^ind^, and B_z_^ind^)., with isosurface values set to ± 3.0 ppm. Color code: blue: shielding; red: deshielding. In addition, the NICS_aniso_ is provided (Isosurface value of 26.0 ppm). Contour plot representation given from side and above views (note the Cartesian framework).

To further assess whether [Zr@Sb_12_]^2−^ exhibits a shielding‐cone response characteristics of aromatic systems [38, 44], the individual components of the induced magnetic field, **B**_i_^ind^ (i = *x*, *y*, *z*), were examined under different orientations of the external magnetic field. When the external field is aligned along the *C*_3_‐symmetry axis, namely the *z*‐axis, the **B**_z_^ind^ map displays a long‐range shielding region passing through the cluster center, accompanied by a complementary perpendicular deshielding contour. This pattern is consistent with a shielding‐cone behavior. The shielding region extends to 8.0 Å above the cluster center at −3.0 ppm and to 11.0 Å at −1.0 ppm. Similar shielding‐cone features are observed when the external field is applied along the perpendicular *C*_2_‐symmetry axis, namely the *x* axis, and along the *y* axis, with shielding of −3.0 ppm extending to approximately 8.0 Å from the cluster center. In these orientations, the complementary deshielding regions are mainly localized near the upper and lower Sb_3_ motifs. The presence of shielding‐cone behavior in multiple field orientations supports the spherical aromatic character of [Zr@Sb_12_]^2−^ and reinforces its description as an integrated aromatic cluster rather than as three discrete Sb_4_ fragments coordinated to a central Zr atom.

Independent component maps from iso‐chemical shielding surface analysis for [Zr@Sb_12_]^2−^ further support this assignment (ICSS, Figure S10). The cluster core is fully enclosed by green shielding isosurfaces, whereas the peripheral yellow deshielding regions display an annular distribution in the XX, YY, and ZZ views. Together, these features form a closed spherical deshielding shell surrounding the cluster center. This radial magnetic pattern, consisting of core shielding and peripheral deshielding, is a hallmark of spherical aromaticity and reflects a delocalized diamagnetic ring‐current response over the Sb_12_ framework.

Incorporation of one additional Sb vertex substantially alters the electronic and magnetic characteristics of the cluster. The optimized [Zr@Sb_13_]^3−^ structure adopts approximate *C*_4v_ symmetry and can be derived from [Zr@Sb_12_]^2−^ by redistribution of the three Sb_4_ units, retaining two parental Sb_4_ fragments that are closed by two Sb_2_ motifs. This arrangement produces a cage‐like Sb_13_ framework encapsulation the central Zr atom, with calculated Zr–Sb distances ranging from 2.834 to 3.324 Å, in good agreement with the crystallographically observed range of 2.802–3.262 Å. Interestingly, the crystal structure displays lower *C*_2v_ symmetry. This experimentally observed structure is calculated to be only 0.45 kcal/mol higher in energy than the optimized *C*_4v_ structure, indicating that the [Zr@Sb_13_]^3−^ cage is sufficiently flexible to be perturbed by counterions and crystal‐packing effects.

The additional Sb atom increases the total valence electron count of [Zr@Sb_13_]^3−^ to 72 electrons, calculated as 4 electrons from Zr, 65 electrons from thirteen Sb atoms, and 3 electrons from the overall charge. AdNDP analysis assigns these electrons first to thirteen Sb 5s lone pairs, represented as 1c–2e elements. In contrast to [Zr@Sb_12_]^2−^, the Zr 4d contribution is distributed over a set of 10c–2e multicenter bonds involving the Zr atom and nine Sb atoms, reflecting more extensive Sb 5p‐Zr 4d orbital mixing within the cage‐like framework (Figure 5). The Sb_13_ framework is further supported by sixteen localized Sb‐Sb 2c–2e bonds, consistent with a connected cage rather than a collection of independent Sb_n_ fragments. The key difference between [Zr@Sb_12_]^2−^ and [Zr@Sb_13_]^3−^arises from the remaining cluster‐delocalized electrons. In [Zr@Sb_13_]^3−^, six electrons are distributed over three 13c–2e delocalized bonding elements involving the tangential Sb 5p orbitals. This corresponds to an S^2^P^4^ superatomic electron configuration, which does not satisfy the 2(*N*+1)^2^ Hirsch rule for 3D aromaticity [22]. Thus, despite its cage‐like structure, [Zr@Sb_13_]^3−^ is not expected to exhibit spherical aromaticity. Orbital‐composition analysis shows that the twelve Sb–Sb 2c–2e bonds in [Zr@Sb_12_]^2−^ contain 95.34% Sb 5p character, whereas the sixteen corresponding bonds in [Zr@Sb_13_]^3−^ contain 92.42%–99.55% Sb 5p character. Thus, the localized Sb–Sb bonds in both clusters arise predominantly from Sb 5p orbitals, with only minor s‐orbital contributions.

**FIGURE 5:**
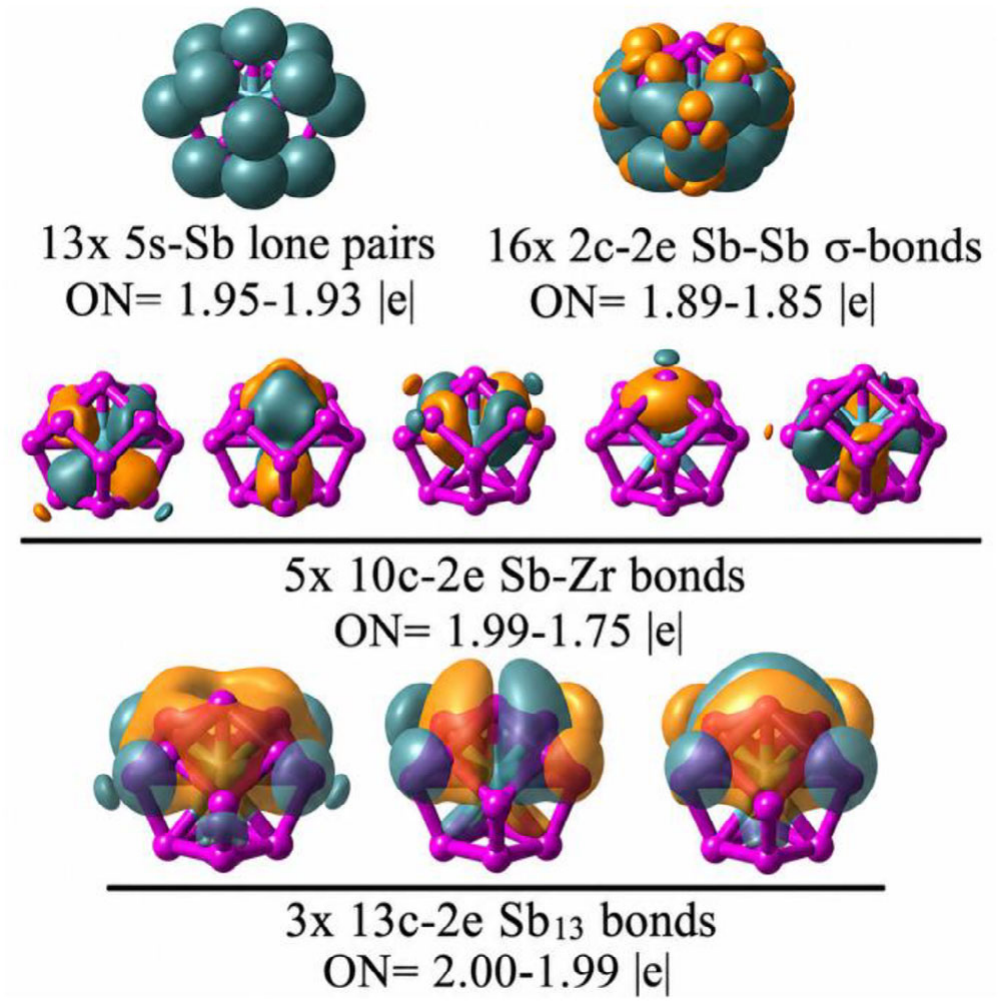
Bonding elements from AdNDP calculations for the [Zr@Sb_13_]^3−^ cluster. ON stands for occupation numbers, given in electrons.

The magnetic response of [Zr@Sb_13_]^3−^ confirms this distinction. The NICS isosurface reveals both shielding and deshielding regions rather than a continuous central shielding domain, in sharp contrast to [Zr@Sb_12_]^2−^ (Figure 6). This behavior is consistent with the nonspherical cluster shape and the incomplete S^2^P^4^ superatomic electron configuration [45]. Component‐resolved induced‐field maps further show orientation‐dependent shielding and deshielding contributions arising from local ring‐like substructures. These local magnetic responses prevent formation of a global shielding cone, resembling the behavior reported for nonaromatic C_60_ fullerene [45, 46]. Therefore, [Zr@Sb_13_]^3−^ displays non‐spherical aromatic behavior that contrasts sharply with the spherical aromaticity of [Zr@Sb_12_]^2−^.

**FIGURE 6:**
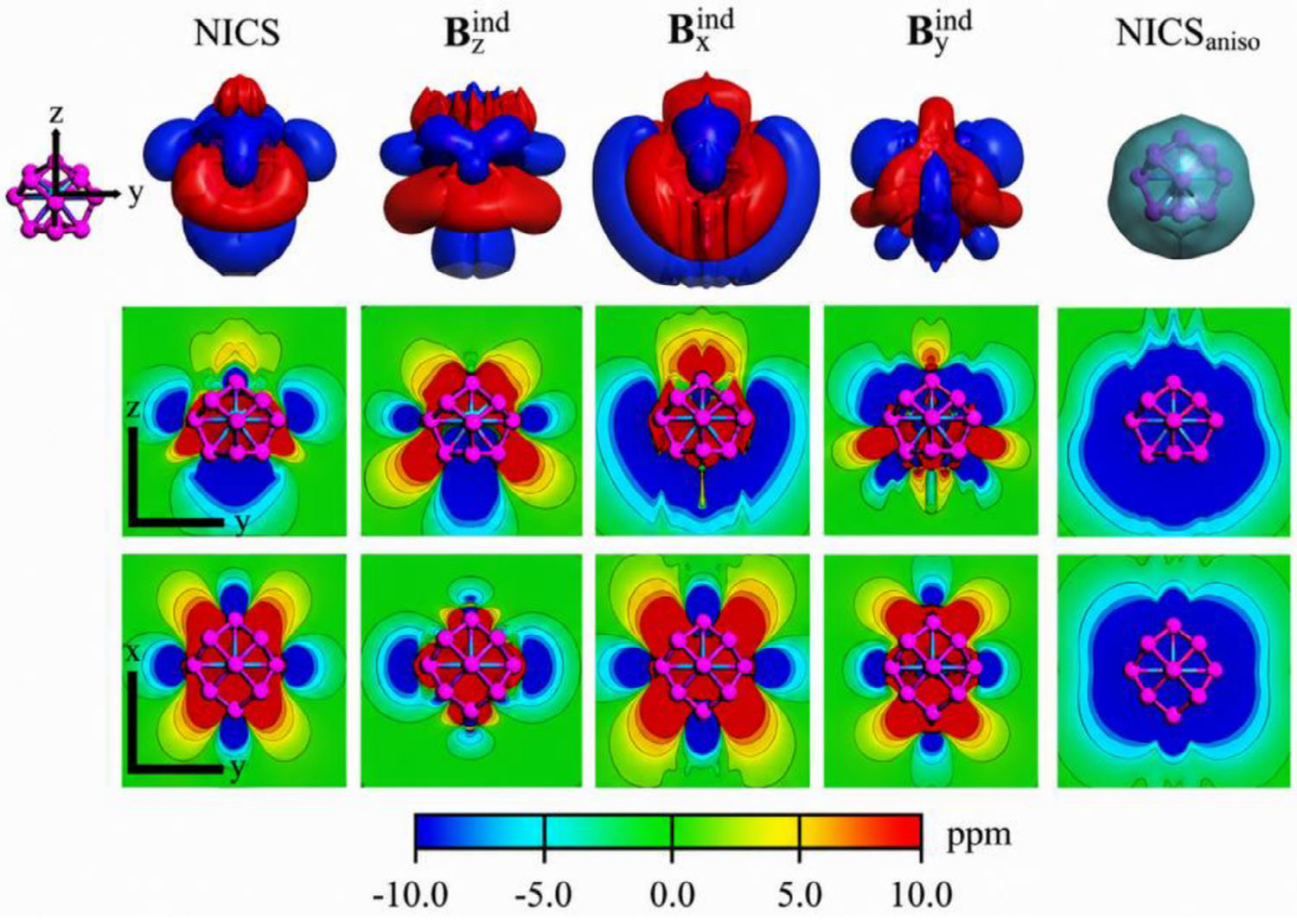
Magnetic response properties for [Zr@Sb_13_]^3−^, accounting for NICS, and under certain representative orientations of the external field (B_x_^ind^, B_y_^ind^, and B_z_^ind^)., with isosurface values set to ±3.0 ppm. Color code: blue: shielding; red: deshielding. In addition, the NICS_aniso_ is provided (Isosurface value of 26.0 ppm). Contour plot representation given from side and above views (note the Cartesian framework).

The ICSS component maps of [Zr@Sb_13_]^3−^ provides additional insight into its anisotropic magnetic character (Figure S11). Along the ZZ direction, the cluster is largely surrounded by green shielding isosurfaces together with a continuous yellow deshielding ring at the outer periphery, indicating that this direction provides the dominant contribution to the aromatic response. By contrast, the deshielding contributions along the XX and YY directions are substantially weaker. These results show that [Zr@Sb_13_]^3−^ does not possess typical spherical aromaticity; instead, it exhibits anisotropic and direction‐dependent aromatic character. The diamagnetic current along the ZZ direction provides the major stabilizing contribution, whereas weak paramagnetic contributions along the XX and YY directions likely arise from the geometric and electronic asymmetry of the Sb_13_ framework.

The anisotropy of the induced magnetic field (Figure 4) was further evaluated to distinguish global spherical aromaticity from possible local aromaticity within the Sb_4_ units. Localized two‐dimensional aromatic units generally produce large anisotropy in the magnetic response [40]. The anisotropy of NICS, NICS_aniso,_ was calculated from the principal components of the shielding tensor according to the standard convention, where NICS_33_>NICS_22_>NICS_11_, using the expression NICS_aniso_ = NICS_33_‐(NICS_11_+NICS_22_)/2 [47]. For [Zr@Sb_12_]^2−^, a sizable anisotropy greater than 26.0 ppm is distributed over the entire cluster and follows the Sb–Sb bonding framework, rather than being localized within individual Sb_4_ units. This result further supports the assignment of [Zr@Sb_12_]^2−^ as a globally delocalized spherical aromatic cluster, rather than a Zr center coordinated by three individually aromatic Sb_4_ ligands. By contrast, [Zr@Sb_13_]^3−^ shows larger and more orientation‐dependent NICS anisotropy (Figure 6), consistent with its nonspherical magnetic response and anisotropic aromatic character.

The electronic structures of [Zr@Sb_12_]^2−^ and [Zr@Sb_13_]^3−^ were further examined by steady‐state UV‐vis absorption spectroscopy in DMF solution. Both clusters display continuous and featureless absorption profiles, with absorbance decreasing monotonically from 300 to 900 nm (Figure 7a). These experimental spectra are well reproduced by TD‐DFT simulations (Figure S12). Density‐of‐states (DOS) analysis shows that the molecular orbitals of both clusters are dominated by Sb contributions (Figure S13a, b). Notably, the Zr contribution to the LUMO of [Zr@Sb_12_]^2−^ is substantially smaller than its contribution to the HOMO, whereas the opposite trend is observed for [Zr@Sb_13_]^3−^ (Figure S14). In [Zr@Sb_12_]^2−^, the intrinsic spherical aromaticity gives rise to a highly delocalized HOMO network, which stabilizes the occupied molecular orbitals [48]. This aromatic stabilization lowers the HOMO energy and contributes to the larger HOMO‐LUMO gap calculated for [Zr@Sb_12_]^2−^ relative to [Zr@Sb_13_]^3−^.

**FIGURE 7:**
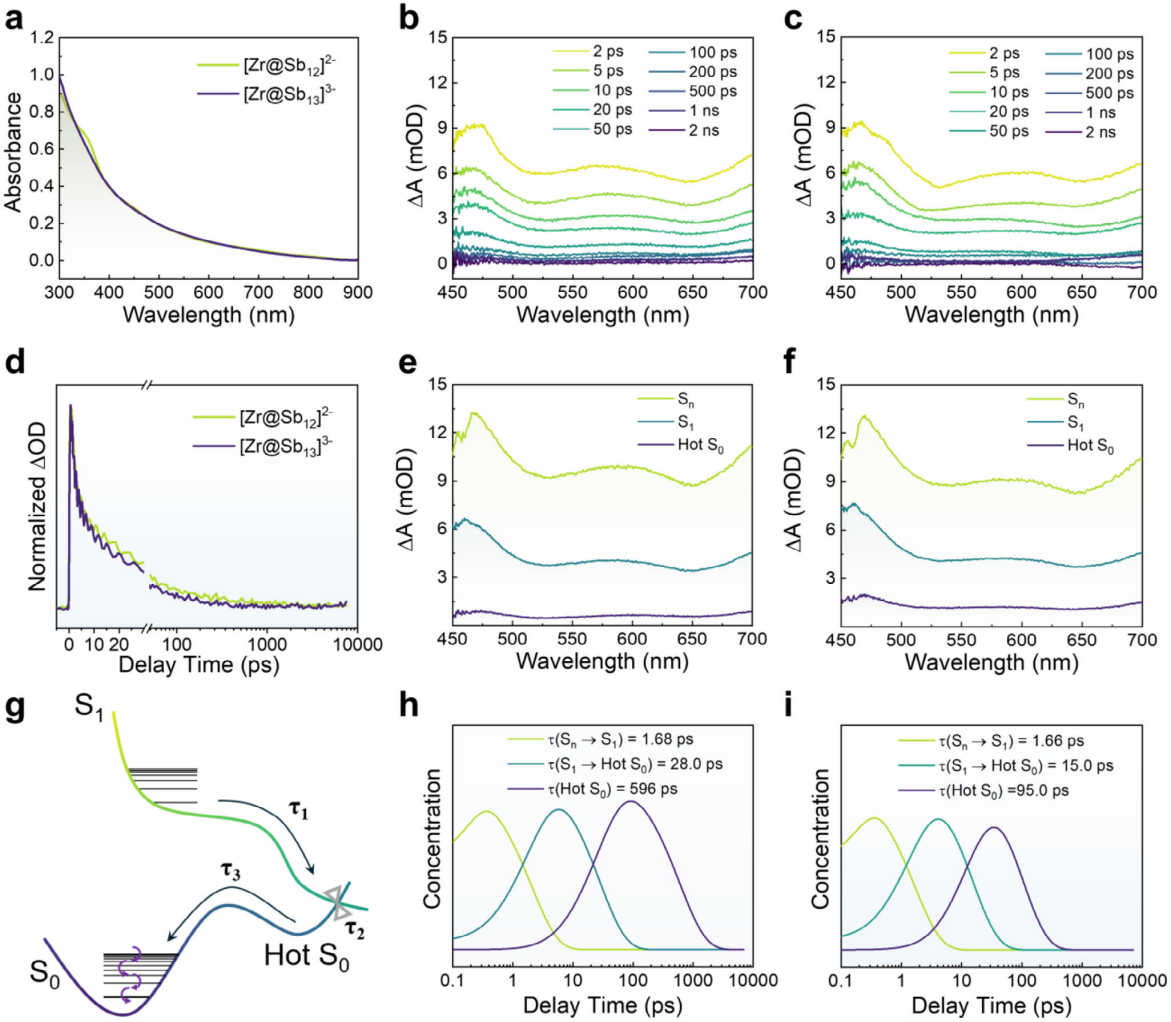
Steady‐state absorption feature and excited‐state dynamic of [Zr@Sb_12_]^2^^−^ and [Zr@Sb_13_]^3^^−^. (a) UV‐vis absorption spectra of [Zr@Sb_12_]^2^^−^ and [Zr@Sb_13_]^3^^−^ in DMF. (b), (c) Femtosecond transient absorption (Fs‐TA) spectra of [Zr@Sb_12_]^2^^−^and [Zr@Sb_13_]^3^^−^ in DMF, respectively, following 400 nm excitation. (**d)** Comparison of the kinetic traces between [Zr@Sb_12_]^2^^−^and [Zr@Sb_13_]^3^^−^ at 600 nm. (e, f) Evolution‐associated different spectra (EADS) obtained from the global lifetime analysis of the fs‐TA data for [Zr@Sb_12_]^2^^−^ and [Zr@Sb_13_]^3^^−^, respectively. (g) Proposed photophysical pathways of [Zr@Sb_12_]^2−^ and [Zr@Sb_13_]^3−^. (h, i) Time‐dependent populations of the transient species obtained from global lifetime analysis for [Zr@Sb_12_]^2−^ and [Zr@Sb_13_]^3−^, respectively.

To determine how aromaticity affects excited‐state dynamics, fs‐TA spectroscopy was used to compare the photophysical behavior of aromatic [Zr@Sb_12_]^2−^ and non‐spherically aromatic [Zr@Sb_13_]^3−^. Upon 400 nm exciation, both clusters exhibit similar TA features, including excited‐state absorption bands centered at approximately 475 nm and 600 nm, together with a broad absorption extending beyond 700 nm. These signals decay within 2 ns (Figure 7b, f). However, the decay kinetics of [Zr@Sb_13_]^3−^ are markedly faster than those of [Zr@Sb_12_]^2−^ (Figure 7e). This kinetic difference is also observed in the fs‐TA spectra under 320 nm excitation (Figures S15 and S16), indicating that the slower relaxation of [Zr@Sb_12_]^2−^ is an intrinsic feature of the cluster rather than an excitation‐wavelength‐dependent effect. Global lifetime analysis of the fs‐TA data yielded three evolution‐associated difference spectra (EADS) components for both clusters. The extracted lifetimes are *τ*_1_ = 1.68 ps, *τ*_2_ = 28.0 ps, and *τ*_3_ = 596 ps for [Zr@Sb_12_]^2−^, and *τ*_1_ = 1.66 ps, *τ*_2_ = 15.0 ps, and *τ*_3_ = 95.0 ps for [Zr@Sb_13_]^3−^ (Figure 7c,d,g,h). These components are assigned sequentially to internal conversion (IC) from the higher‐lying excited states S_n_ to the S_1_ state [49], rapid IC from the S_1_ state to the ground state S_0_, and subsequent relaxation of the hot ground state [50, 51, 52]. The nearly identical *τ*_1_ values suggests that early relaxation of from S_n_ to S_1_ is relatively insensitive to the aromatic difference between the two clusters. In contrast, the substantially longer *τ*_2_ and *τ*_3_ lifetimes of [Zr@Sb_12_]^2−^ indicate that spherical aromaticity slows both S_1_ to S_0_ internal conversion and hot‐ground‐state relaxation. This difference can be rationalized by the combined electronic and structural consequences of aromatic stabilization. The spherical aromaticity of [Zr@Sb_12_]^2−^ enlarges the energy gap, thereby suppressing nonradiative decay according to the energy‐gap law [53, 54, 55, 56], and also rigidifies the Sb_12_ framework, reducing access to low‐frequency vibrational modes that facilitate structural relaxation [57]. As a result, aromatic stabilization in [Zr@Sb_12_]^2−^ significantly decelerates both internal conversion and subsequent relaxation processes relative to the non‐spherically aromatic [Zr@Sb_13_]^3−^ cluster.

Theoretical calculations further support the influence of aromaticity on the optical and excited‐state properties. The dominant S_0_ → S_n_ transitions in both clusters involve hole‐electron distributions delocalized over the entire [Zr@Sb_12_]^2−^ and [Zr@Sb_13_]^3−^ framework (Figure 8), with contributions from Zr 4d orbitals and radial orbitals of the antimony cage. Thus, the optical transitions are directly associated with the cluster orbitals that define the aromatic or non‐spherical aromatic character. Optimization of the S_1_‐state geometries further reveals that [Zr@Sb_12_]^2−^ undergoes only minor structural distortion relative to its S_0_ geometry, with a root‐mean‐square deviation (RMSD) of 0.10 Å. This distortion mainly involves a slight shortening of the selected Zr‐Sb distances from 3.333 to 3.216 Å. By contrast, [Zr@Sb_13_]^3−^ shows a larger S_1_‐state distortion, with an RMSD of 0.17 Å, primarily associated with elongation of selected Sb–Sb distances at the bottom of the cage from 2.863 to 3.438 Å. These calculations indicate that the spherical aromatic [Zr@Sb_12_]^2−^ framework is structurally more rigid in the excited state, whereas addition of a single Sb atom to form [Zr@Sb_13_]^3−^ disrupts spherical aromaticity, enhances structural flexibility, and accelerates nonradiative relaxation.

**FIGURE 8:**
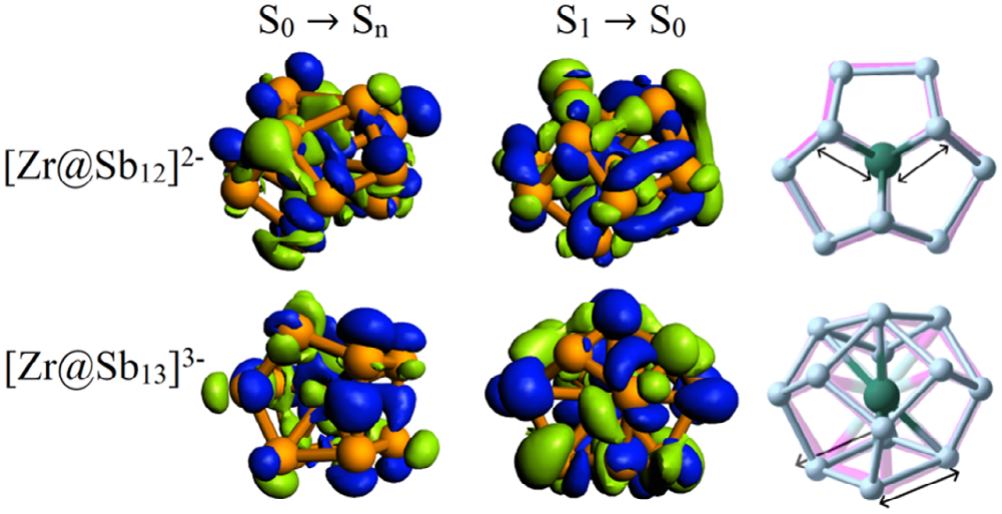
Calculated electron‐hole pairs ascribed to the initial S_0_ → S_n_ excitation and subsequent S_1_ → S_0_ radiative decay, with blue and green isosurfaces representing holes and electrons. In addition, the S_0_ (pink) and S_1_ (grey) structures are given denoting main geometrical distortions by black arrows.

## 3 Conclusion

In summary, we have isolated and characterized two endohedral zirconium–antimony Zintl clusters, [Zr@Sb_12_]^2−^ and [Zr@Sb_13_]^3−^, that provide a rare atom‐by‐atom comparison of structural evolution, aromaticity, and excited‐state dynamics in all‐metal superatoms. Structural and bonding analyses reveal that [Zr@Sb_12_]^2−^ behaves as an integrated Sb_12_ cluster framework rather than as three discrete Sb_4_ rings coordinated to a central Zr atom. The delocalized bonding pattern, supported by AdNDP and magnetic‐response analyses, gives rise to a closed‐shell S^2^P^6^ superatomic configuration and pronounced spherical all‐metal aromaticity. Comparison with the related [La(η^4^‐Sb_4_)_3_]^3−^ cluster further highlights the decisive role of the encapsulated transition‐metal center in controlling the geometry, electron count, and extent of delocalization within the Sb_12_ framework.

The one‐atom‐expanded [Zr@Sb_13_]^3−^ cluster demonstrates how sensitively superatomic aromaticity depends on nuclearity and framework symmetry. Incorporation of a single additional Sb atom reorganizes the antimony framework, disrupts superatomic shell closure, and produces an S^2^P^4^ electronic configuration with markedly attenuated spherical aromaticity. Thus, a minimal change in composition leads to a substantial change in electronic structure, establishing atomically precise nuclearity as a key parameter for regulating aromaticity in endohedral Zintl clusters.

Importantly, the consequences of this aromaticity modulation extend beyond ground‐state stabilization. Fs‐TA spectroscopy shows that the spherically aromatic [Zr@Sb_12_]^2−^ cluster exhibits significantly slower excited‐state relaxation than the less aromatic [Zr@Sb_13_]^3−^ analogue. This difference is attributed to the larger electronic energy gap and greater structural rigidity associated with aromatic stabilization in [Zr@Sb_12_]^2−^. These results establish a direct structure–aromaticity–dynamics relationship, demonstrating that 3D aromaticity can govern not only the stability of all‐metal clusters in the ground state, but also their excited‐state robustness. More broadly, this work provides a molecular‐level design principle for tuning the electronic and photophysical properties of superatomic Zintl clusters through single‐atom structural control.

## Author Contributions

Zhong‐Ming Sun and Teng‐Teng Chen conceived the project and designed the experiments. Yun Zhang conducted the synthesis. Yun Zhang and Ziqi Deng completed the experimental validation and result testing. Wen‐Juan Tian and Alvaro Muñoz‐Castro performed the quantum chemical calculations and analyzed the data. Zhong‐Ming Sun, Yun Zhang, Alvaro Muñoz‐Castro, Wen‐Juan Tian, Ziqi Deng, and Teng‐Teng co‐wrote the manuscript. All authors reviewed the paper.

## Conflicts of Interest

The authors declare no conflicts of interest.

## Supporting information



The Supporting information is available in the online version of the paper. Crystallographic data for the structures reported in this Article have been deposited at the Cambridge Crystallographic Data Centre under deposition numbers CCDC 2543586 (for **1**) and CCDC 2543587 (for **2**), which contain the supplementary crystallographic data for this paper. These data can be obtained free of charge from The Cambridge Crystallographic Data Centre via https://www.ccdc.cam.ac.uk/structure. **Supporting File**: anie74038‐sup‐0001‐SuppMat.docx.

## Data Availability

The data that supports the findings of this study are available in the Supporting Information of this article
