# Supplementary material for: Single‐Atom Control of Aromaticity and Excited‐State Dynamics in Endohedral Zirconium–Antimony Zintl Clusters
Source: Angew Chem Int Ed Engl. 2026 Aug 6;65(41):e6088506. doi: 10.1002/anie.6088506 (PMC13638084; doi:10.1002/anie.6088506)
Supplement: Supplementary file 1 [file ANIE-65-e6088506-s001.docx]

**Single-Atom Control of Aromaticity and Excited-State Dynamics in Endohedral Zirconium–Antimony Zintl Clusters**

Yun Zhang ^[a,g]^ , Ziqi Deng^[b,g]^, Wen-Juan Tian^[c,g]^, Hui-Qing Sun^[c]^, Kang Sun^[b]^, Alvaro Muñoz-Castro*^[d]^, Zhong-Ming Sun*^[a, e]^ & Teng-Teng Chen*^[b,f]^

**Content**

**1: Experimental Procedures..............................................................................................................................2
2: Crystallographic Supplementary Information.............................................................................................5
3: Energy Dispersive X-ray (EDX) Spectroscopic Analysis ………………………………………. ..................11**
**4: Quantum Chemical Calculations................................................................................................................13**

**5: Femtosecond Transient Absorption Spectroscopy .................................................................................16**

1. Experimental Procedures

All manipulations and reactions were performed under a nitrogen atmosphere using standard Schlenk or glovebox techniques. en (ethylenediamine, Aldrich, 99.8%), was freshly distilled by CaH_2_ prior to use and stored in N_2_. toluene (Aldrich, 99.8%), Hex (Hexane, Aldrich, 99.8%), ZrCp_2_Cl_2_ (Aldrich, 98%) was distilled from sodium/benzophenone under nitrogen and stored under N_2_. 2.2.2-crypt (4,7,13,16,21,24-Hexaoxa-1,10 diazabicyclo (8.8.8) hexacosane, acquired from Sigma-Aldirich, 98%) was dried in vacuum for one day prior to use. K_2_Sb and K_5_Sb_4_ was prepared according to literatures methodology ^1^ by heating a stoichiometric mixture of the elements (K: 604 mg, Sb: 778 mg) and (K: 604 mg, Sb: 1506 mg) at 800 °C for 36 h in a niobium tube sealed in evacuated quartz ampules.

**Synthesis of** [K([2.2.2]-crypt)]_2_[Zr@Sb_12_] (**1**)**:**

Method 1

K_2_Sb (60 mg, 0.30 mmol) and 2.2.2-crypt (188 mg, 0.50 mmol) were dissolved in 4.0 mL of en and stirred for 20 minutes, affording a brown solution. In a second vial, ZrCp_2_Cl_2_ (29 mg, 0.10 mmol) was dissolved in 0.5 mL of toluene. The toluene solution was added dropwise to the en solution, generating a brown-green suspension. The reaction mixture was stirred at room temperature for 5 h, during which the suspension gradually changed from green to brown-red. The mixture was then filtered through glass wool and transferred to a test tube, followed by careful layering with toluene (3.5 mL) to promote crystallization. After two weeks, small black needle-shaped crystals of [K([2.2.2]-crypt)]_2_[Zr@Sb_12_] (**1**) were obtained in an overall crystalline yield of approximately 11%, based on the K_2_Sb precursor.

Method 2

K_5_Sb_4_ (68 mg, 0.10 mmol) and 2.2.2-crypt (150 mg, 0.40 mmol) were dissolved in 4.0 mL of en and stirred at 70 °C for 20 min, affording a brown-red solution. In a second vial, ZrCp_2_Cl_2_ (29 mg, 0.10 mmol) was dissolved in 0.5 mL of toluene. The toluene solution was then added dropwise to the en solution, producing a brown-green suspension. The reaction mixture was stirred at room temperature for an additional 5 h, during which the suspension gradually changed to a brown-red color. The mixture was subsequently filtered through glass wool and transferred to a test tube, followed by careful layering with toluene (3.5 mL) to promote crystallization. After three weeks, some black needle-shaped crystals of [K([2.2.2]-crypt)]_2_[Zr@Sb_12_] (**1**) were isolated in an overall crystalline yield of approximately 13%, based on the K_5_Sb_4_ precursor.

**Synthesis of** [K([2.2.2]-crypt)]_3_[Zr@Sb_13_] (**2**)**:**

Method 1

K_5_Sb_4_ (68 mg, 0.10 mmol) and 2.2.2-crypt (150 mg, 0.40 mmol) were dissolved in 4.0 mL of en and stirred at 70 °C for 20 min, affording a brown-red solution. In a second vial, ZrCp_2_Cl_2_ (29 mg, 0.10 mmol) was dissolved in 0.5 mL of toluene. The toluene solution was then added dropwise to the en solution, producing a brown-green suspension. The reaction mixture was stirred at 60 °C for an additional 3 h, during which the suspension gradually changed to a dark brown color. The mixture was subsequently filtered through glass wool and transferred to a test tube, followed by careful layering with toluene (3.5 mL) to promote crystallization. After three weeks, small black block-shaped crystals of [K([2.2.2]-crypt)]_3_[Zr@Sb_13_] (**2**) were isolated in an overall crystalline yield of approximately 26%, based on the K_5_Sb_4_ precursor.

Method 2

Crystals of [K([2.2.2]-crypt)]_2_[Zr@Sb_12_] (**1**) (10 mg) and an additional portion of K_5_Sb_4_ (17 mg, 0.025 mmol) were introduced into en, and the mixture was heated at 60 °C with continuous stirring for another 1 h. During this process, the color of the reaction mixture gradually changed to dark brown-black. The resulting mixture was filtered through glass wool and transferred into a test tube, after which toluene (3.5 mL) was carefully layered on top to promote crystallization. After standing for 2 weeks, black block-shaped crystals of [K([2.2.2]-crypt)]_3_[Zr@Sb_13_] (**2**) were observed at the solution interface on the wall of the test tube. Compound **2** was obtained in a very low crystalline yield of less than 5% based on precursor **1**.

**X-ray Diffraction:**

Suitable single crystals were selected for X-ray diffraction analyses. The single crystal diffraction data were collected on Rigaku XtalAB PRO MM007 DW at 100 K, with a Hybrid Pixel Array CCD Detector. The X-ray sources are Mo Kα (λ = 0.71073 Å) for **1** and Cu Kα(λ = 1.54184 Å) for **2**. The raw diffraction data were collected and reduced by the program of “CrysAlisPro 1.171.40.84a (Rigaku OD, 2020)”, and treated by multi-scan absorption correction. Structures were solved using direct methods and refined using SHELXL-2014 and Olex2 2-4 to convergence, in which all non-hydrogen atoms were refined anisotropically during the final cycles. All hydrogen atoms of the organic molecule were placed by geometrical considerations and were added to the structure factor calculation. Crystallographic data for the structures reported in this paper have been deposited with the Cambridge Crystallographic Data Centre. A summary of the crystallographic data for the compound **1** and **2** are listed in **Table S1** and **S2**, and selected bond distances are given in Table S3. CCDC entries **2543586** and **2543587** for compound **1** and compound **2** contain the supplementary crystallographic data for this paper. These data can be obtained free of charge from <https://www.ccdc.cam.ac.uk/structures/>.

**Energy Dispersive X-ray (EDX) Spectroscopic Analysis:**

EDX analysis on the title cluster was performed using a scanning electron microscope (FE-SEM, JEOL JSM-7800F, Japan). Data acquisition was performed with an acceleration voltage of 15 kV and an accumulation time of 60 s.

**Quantum chemical methods:**

Relativistic density functional theory calculations were carried out by using the ADF2024 code,^[2]^ incorporating scalar corrections via the ZORA Hamiltonian.^[3]^ Triple-§ All-electron (CORE NONE) Slater basis set, plus two polarization functions (STO-TZ2P) quality basis sets were employed, within the hybrid PBE0 functional.^[4]^ Geometry optimizations were performed without symmetry restraint via the analytical energy gradient method implemented by Versluis and Ziegler.^[5]^ An energy convergence criterion of 10^-4^ Hartree, gradient convergence criteria of 10^-3^ Hartree/Å, and radial convergence criteria of 10^-2^ Å were employed to evaluate the relaxed structures. Solvent and counterion effects were taken into account via the conductor-like screening model (COSMO), which uses DMF as a solvent.^[6]^ London dispersion corrections were included using the pairwise correction from Grimme and Becke and Johnson (BJ) damping functions (DFT-D3(BJ)).^[7]^ Calculations of the magnetic behavior was based on a three-dimensional array of nucleus-independent shielding tensor (NICS)^[8-10]^ within the GIAO formalism, by using the ALLINONE keyword from NMR module from ADF2024.^[11,12]^ The resulting induced field (**B**^ind^) upon an external magnetic field (**B**^ext^), obtained according to **B***_i_*^ind^ = -$\text{σ}$*_ij_***B***_j_*^ext^,^[8,10, 13-15]^ where the isotropic term (**B**_iso_^ind^ =-($\text{σ}$_11_+$\text{σ}$_22_+$\text{σ}$_33_)/3) is related to NICS.^[16]^ For convenience, the *i* and *j* suffixes are related to the *x*-, *y*-, and *z*-axes of the Cartesian system (*i,j* = x,y,z). The values of **B**^ind^ are given in ppm in relation to **B**^ext^.^[9,10]^ Adaptive Natural Density Partitioning (AdNDP) analysis^[17,18]^ was carried out to elucidate the chemical bonding, performed from Gaussian 09 outputs at the PBE0-D3(BJ)/Def2-TZVP level of theory,^[19]^ as implemented in the Multiwfn suite.^[20,21]^ All ICSS-related shielding tensor calculations were carried out in the Gaussian 09 program package,^[19]^ employing the PBE0 hybrid functional combined with the def2-TZVP basis set.^[22-24]^ The calculation results were analyzed using the Multiwfn program^[21]^ to extract the isotropic component and the anisotropic shielding data along the XX, YY, and ZZ directions. Finally, the magnetic shielding isosurfaces were visually presented via the VMD 1.9.3 software.^[25]^ The simulated UV-vis absorption spectra and the Kohn-Sham orbital were calculated by Gaussian 16 program package^[19]^ based on PBE0-D3(BJ)/Def2-TZVP level of theory and the frontier orbital was drawn using the VMD 2.0.^[26]^ The density of state (DOS) was analyzed by Multiwfn program.^[21]^

**Steady-State UV-vis Absorption Spectroscopy:**

The sample was prepared into acetonitrile solution and the UV-vis spectrum was measured by the UV-2600 UV-vis absorption spectrometer (Shimadzu, Japan).

**Femtosecond Transient Absorption Spectroscopy:**

The fs-TA measurements were conducted using the *Helios* pump-probe transient absorption spectrometer system (Ultrafast Systems, USA) with the femtosecond laser from the *Astrella USP* regenerative amplified Ti:sapphire laser system (Coherent, USA). The 800-nm laser light with 34-fs pulse width was subsequently split into two beams, one as the pump beam and another one as the probe beam. The pump beam passed through an *Apollo-T* optical parametric amplifier (Ultrafast Systems, USA) to generate the 320-nm and 400-nm pump beam, whereas the probe beam passed through a sapphire crystal and generated a white-light continuum (450-800 nm). The time-delayed probe beam was controlled by the optical delay rail with a maximum temporal delay at 8 ns. Two beams pass through the samples, and the signals were then collected by the detector. The nanoclusters were dissolved in the anhydrated DMF under the N_2_ atmosphere, and the sample solutions were measured in a 2-mm path-length quartz cuvette. The spectrometric data were recorded three times for each sample in a 3D wavelength-time-absorbance matrix with the average time of 2 seconds.

2. Crystallographic Supplementary information

Table S1. X-ray measurements and structure solutions of compound 1

| Compound | [K([2.2.2]-crypt)]_2_[Zr@Sb_12_] |
| --- | --- |
| Empirical formula | C_43_H_77_K_2_N_4_O_12_Sb_12_Zr |
| Formula weight | 2472.50 |
| Crystal system | triclinic |
| Space group | *P*$\bar{\text{1}}$ |
| *a* /Å | 15.5597(3) |
| *b* /Å | 16.5402(3) |
| *c* /Å | 16.5488(3) |
| *α*/° | 119.020(2) |
| *β*/° | 107.024(2) |
| *γ*/° | 90.9640(10) |
| *V* /Å^3^ | 3496.24(13) |
| *Z* | 2 |
| *ρ*_calc_ /g·cm^-3^ | 2.349 |
| *μ*/mm^-1^ | 4.864 ( Mo Kα) |
| *F* (000) | 2298.0 |
| 2Θ range /° | 5.632 to 61.94 |
| Reflections collected / unique | 72366 / 18498 |
| Data / restraints / parameters | 18498/ 72/ 668 |
| *R*_1_/*wR*_2_ (I>2σ(I)) a | 0.0270; 0.0615 |
| *R*_1_/*wR*_2_ (all data) | 0.0358; 0.0648 |
| GooF (all data) b | 1.025 |
| Data completeness | 0.998 |
| Max. peak/hole /e-·Å-3 | 0.961/-0.866 |

*^a^* *R*_1_= = ∑||F_o_|-|F_c_||/∑|F_o_|; *wR*_2_ = {∑*w*[(F_o_)^2^-(F_c_)^2^]^2^/∑*w*[(F_o_)^2^]^2^}^1/2^

*^b^* GooF = {∑w[(Fo)^2^-(Fc)^2^]^2^/(n-p)}^1/2^

**Table S2.** X-ray measurements and structure solutions of compound **2**

| Compound | [K([2.2.2]-crypt)]_3_[Zr@Sb_13_] |
| --- | --- |
| Empirical formula | C_61_H_116_K_3_N_6_O_18_Sb_13_Zr |
| Formula weight | 3012.86 |
| Temperature/K | 100(2) |
| Crystal system | monoclinic |
| Space group | *P*2_1_/*m* |
| a/Å | 14.3971(2) |
| b/Å | 21.8917(3) |
| c/Å | 16.2587(3) |
| *α*/° | 90 |
| *β*/° | 107.073(2) |
| *γ*/° | 90 |
| *V* /Å^3^ | 4898.54(14) |
| Z | 2 |
| *ρ*_calc_ /g·cm^-3^ | 2.043 |
| *μ*/mm‑1 | 30.338 (Cu Kα) |
| F(000) | 2856.0 |
| *μ*/mm^-1^ | Cu Kα(λ = 1.54184) |
| 2Θ range /° | 6.422 to 133.202 |
| Reflections collected / unique | 50793/8891 |
| Data / restraints / parameters | 8891/802/ 599 |
| *R*_1_/*wR*_2_ (I>2σ(I)) a | 0.0801; 0.2269 |
| *R*_1_/*wR*_2_ (all data) | 0.0869; 0.2359 |
| GooF (all data) b | 1.045 |
| Data completeness | 0.999 |
| Max. peak/hole /e-·Å-3 | 4.017/-2.249 |

*^a^* *R*_1_= = ∑||F_o_|-|F_c_||/∑|F_o_|; *wR*_2_ = {∑*w*[(F_o_)^2^-(F_c_)^2^]^2^/∑*w*[(F_o_)^2^]^2^}^1/2^

*^b^* GooF = {∑w[(Fo)^2^-(Fc)^2^]^2^/(n-p)}^1/2^

**Table S3.** Selected bond lengths (Sb‒Sb and Zr–Sb bond) from crystallographic and DFT-optimized structures (PBE0 functional) for compound **1** and **2**

|  | | |  | | |
| --- | --- | --- | --- | --- | --- |
| Bonds | Experimental length | DFT length | Bonds | Experimental length | DFT length |
| Sb1–Sb2 | 2.8223 | 2.86379 | Sb1-Sb2 | 3.0871 | 3.118311 |
| Sb1–Sb3 | 2.8381 | 2.86379 | Sb1-Sb3 | 3.0871 | 3.118311 |
| Sb1–Zr4 | 2.9393 | 2.93482 | Sb1-Sb4 | 3.0871 | 3.118311 |
| Sb10–Sb6 | 2.8152 | 2.86379 | Sb1-Sb5 | 3.0871 | 3.118311 |
| Sb10–Sb9 | 2.8053 | 2.86379 | Sb1-Zr6 | 2.8020 | 2.857624 |
| Sb10–Zr4 | 2.9725 | 2.93482 | Sb2-Zr6 | 3.2038 | 3.39825 |
| Sb11–Sb13 | 2.8028 | 2.86379 | Sb2-Sb7 | 2.8381 | 2.814531 |
| Sb11–Sb7 | 2.8297 | 2.86379 | Sb2-Sb8 | 2.8023 | 2.814531 |
| Sb11–Zr4 | 2.959 | 2.93482 | Sb3-Zr6 | 3.2120 | 3.39825 |
| Sb12–Sb13 | 2.8287 | 2.86379 | Sb3-Sb7 | 2.8256 | 2.814531 |
| Sb12–Sb7 | 2.8121 | 2.86379 | Sb3-Sb9 | 2.7974 | 2.814531 |
| Sb12–Zr4 | 2.9455 | 2.93482 | Sb4-Zr6 | 3.2038 | 3.39825 |
| Sb13–Sb9 | 2.9552 | 2.85017 | Sb4-Sb8 | 2.8020 | 2.814531 |
| Sb13–Zr4 | 3.3531 | 3.33203 | Sb4-Sb10 | 2.8381 | 2.814531 |
| Sb2–Sb5 | 2.8131 | 2.86379 | Sb5-Zr6 | 3.2120 | 3.39825 |
| Sb2–Sb6 | 2.9548 | 2.85017 | Sb5-Sb9 | 2.7974 | 2.814531 |
| Sb2–Zr4 | 3.3255 | 3.33203 | Sb5-Sb10 | 2.8256 | 2.814531 |
| Sb3–Sb5 | 2.8129 | 2.86379 | Zr6-Sb7 | 3.4059 | 3.331127 |
| Sb3–Sb7 | 2.9502 | 2.85017 | Zr6-Sb8 | 3.2181 | 3.331127 |
| Sb3–Zr4 | 3.3682 | 3.33203 | Zr6-Sb9 | 3. 2181 | 3.331127 |
| Sb5–Zr4 | 2.9774 | 2.93482 | Zr6-Sb10 | 3.4059 | 3.331127 |
| Sb6–Sb8 | 2.8146 | 2.86379 | Zr6-Sb11 | 3.1126 | 3.117927 |
| Bonds | Experimental length | DFT length | Bonds | Experimental length | DFT length |
| Sb6–Zr4 | 3.3218 | 3.33203 | Zr6-Sb12 | 3.2869 | 3.117927 |
| Sb7–Zr4 | 3.3689 | 3.33203 | Zr6-Sb13 | 3.1126 | 3.117927 |
| Sb8–Sb9 | 2.8346 | 2.86379 | Zr6-Sb14 | 3.2869 | 3.117927 |
| Sb8–Zr4 | 2.9554 | 2.93482 | Sb7-Sb11 | 2.8259 | 2.765385 |
| Sb9–Zr4 | 3.3305 | 3.33203 | Sb8-Sb12 | 2.8550 | 2.765385 |
|  |  |  | Sb9-Sb13 | 2.8259 | 2.765385 |
|  |  |  | Sb10-Sb14 | 2.8550 | 2.765385 |
|  |  |  | Sb11-Sb12 | 2.8714 | 3.091863 |
|  |  |  | Sb11-Sb13 | 2.8714 | 3.091863 |
|  |  |  | Sb12-Sb14 | 2.8710 | 3.091863 |
|  |  |  | Sb13-Sb14 | 2.8710 | 3.091863 |
|  |  |  |  |  |  |

**Figure S1.** Crystals of compound **1** and **2**

**Figure S2.** Asymmetric unit of (**1**). Thermal ellipsoids are set at 50% probability. Hydrogen atoms have been omitted for clarity.

**Figure S3.** Unit cell of (**1**). Thermal ellipsoids are set at 50%probability. Hydrogen atoms have been omitted for clarity.

**Figure S4.** Asymmetric unit of (**2**). Thermal ellipsoids are set at 50% probability. Hydrogen atoms have been omitted for clarity.

**Figure S5.** Unit cell of (**2**). Thermal ellipsoids are set at 50% probability. Hydrogen atoms have been omitted for clarity.

3. Energy Dispersive X-ray (EDX) Spectroscopic Analysis

The Energy-Dispersive X-ray Spectroscopy (EDX) results for compounds **1** and **2** are presented in Figures S6–S7. The measured atomic ratios of zirconium (Zr) to antimony (Sb) are 1.3/11.4 and1.4/12.8 respectively. These values are generally consistent with those derived from single-crystal X-ray diffraction analysis. Minor deviations observed in the EDX results for all samples can be attributed to the irregular crystal surfaces caused by prolonged exposure to air. It should also be noted that the quantitative accuracy for potassium (K) is limited, and therefore, the EDX data should be regarded as qualitative.

| Coumpand **1** | Element | wt% | σ | Experimental / Calculated Atom |
| --- | --- | --- | --- | --- |
|  | K | 4.9 | 0.7  4 | 2/2 |
|  | Sb | 87.4 | 0.7 | 11.5/12 |
|  | Zr | 7.6 | 0.3 | 1.3/1 |

**Figure S6**. EDX analysis of compound **1**.

| Coumpand **2** | Element | wt% | σ | Experimental / Calculated Atom |
| --- | --- | --- | --- | --- |
|  | K | 7.4 | 0.3  4 | 3.4/3 |
|  | Sb | 85.4 | 0.6 | 12.8/12 |
|  | Zr | 7.2 | 0.6 | 1.4/1 |

**Figure S7**. EDX analysis of compound **2**.

4. **Quantum Chemical Calculations**

**Table S4.** Energy difference (ΔΔE) between the *D*_3h_ and *I*_h_ isomers for [Zr@Sb_12_]^2-^, accounting for the different contributions to the formation energy (ΔE_int_), given by Pauli repulsion (ΔE_pauli_), electrostatic character (ΔE_elstat_), orbital overlap (ΔE_orb_), and London dispersion contributions (ΔE_disp_). Values in kcal/mol. Note that the larger difference in energy is provided by the ΔE_orb_ term, with an energy difference of ΔΔE_orb_ = 226.9 kcal/mol, denoting that the *I*_h_ isomer exhibits a sizably lower orbital interaction stabilization (orbital overlap).

|  | *D*_3h_ | *I*_h_ | ΔΔE |
| --- | --- | --- | --- |
|  |  |  |  |
| ΔE_pauli_ | 3867.1 | 3808.9 | -58.1 |
| ΔE_elstat_ | -1849.2 | -1868.8 | -19.5 |
| ΔE_orb_ | -3658.2 | -3431.3 | 226.9 |
| ΔE_disp_ | -50.1 | -52.6 | -2.5 |
|  |  |  |  |
| ΔE_int_ | -1690.4 | -1543.8 | 146.8 |

**Figure S8.** Determination of the bowl-depth for the Sb_4_ units by measuring the distance between alternate Sb-Sb centroids (green dots), for [Zr@Sb_12_]^2-^ and [La(*η*^4^-Sb_4_)_3_]^3-^.

**Figure S9.** Suggested shift from [Zr@Sb_12_]^2-^ to [Zr@Sb_13_]^3-^ by incorporation of an extra Sb atom. The atoms remaining at the same positions are given in white, while the moving atoms numbered in yellow.

**Figure S10.** Calculated ICSSs of [Zr@Sb_12_]^2-^. Top and side views of ICSSXX/ICSSYY/ICSSZZ. The yellow and green regions correspond to negative and positive ICSS values, respectively. ICSSXX/YY/ZZ isosurface = ±3 ppm.

**Figure S11.** Calculated ICSSs of [Zr@Sb_13_]^3-^. Top and side views of ICSSXX/ICSSYY/ICSSZZ. The yellow and green regions correspond to negative and positive ICSS values, respectively. ICSSXX/YY/ZZ isosurface = ±3 ppm.

The extent and spatial distribution of electron delocalization were further quantified using the electron density of delocalized bonds (EDDB) method (Figure S12). ^[27–29]^ For [Zr@Sb_12_]^2-^, the global component, EDDB_G_, contains 14.9 |*e*|, of which 12.8 |*e*| are assigned primarily to the Sb_12_ rim (EDDB_F_). This concentration of delocalized density within the Sb_12_ framework is consistent with the magnetic-response analysis. For [Zr@Sb_13_]^3-^, EDDB_G_ contains 25.0 |*e*|, whereas 16.2 |*e*| are associated with the Sb_13_ cage (EDDB_F_), indicating a larger contribution from delocalization involving the remaining cluster framework than in [Zr@Sb_12_]^2−^.

**Figure S12.** Electron density of delocalized bonds (EDDB) isosurfaces for the global [Zr@Sb_12_]^2-^ and [Zr@Sb_13_]^3-^ clusters (EDDB_G_) and their corresponding Sb_12_ and Sb_13_ framework (EDDB_F_), shown in two orientations. Isosurface value set at 0.02 a.u.

**5. Steady-State UV-vis and Femtosecond Transient Absorption Spectroscopy**

**Figure S13.** TD-DFT simulated UV-vis absorption spectra of **a** [Zr@Sb_12_]^2^⁻and **b** [Zr@Sb_13_]^3^⁻.

**Figure S14.** Density of state for **a** [Zr@Sb_12_]^2^⁻and **b** [Zr@Sb_13_]^3^⁻. Kohn–Sham molecular orbital energy levels diagram and atomic orbital components for **c** [Zr@Sb_12_]^2^⁻and **d** [Zr@Sb_13_]^3^⁻.

**Figure S15.** Frontier molecular orbital for [Zr@Sb_12_]^2^⁻and [Zr@Sb_13_]^3^⁻.

**Figure S16. a** Fs-TA spectra of [Zr@Sb_12_]^2^⁻in DMF following 320 nm excitation. **b** EADS obtained by global analysis of the fs-TA data for [Zr@Sb_12_]^2^⁻. **c** Time-dependent populations of the transient species derived from the global analysis of [Zr@Sb_12_]^2^⁻. **d** Fs-TA spectra of [Zr@Sb_13_]^3^⁻ in DMF following 320 nm excitation. **e** EADS obtained by global analysis of the fs-TA data for [Zr@Sb_13_]^3^⁻. **f** Time-dependent populations of the transient species derived from the global analysis of [Zr@Sb_13_]^3^⁻.

**Figure S17.** Kinetic traces at 600 nm comparison between [Zr@Sb_12_]^2^⁻ and [Zr@Sb_13_]^3^⁻ in DMF solution upon 320 nm excitation.

**Figure S18.** Kinetic traces and corresponding global fitting curve at **a** 475 nm **b** 600, and **c** 690 nm for [Zr@Sb_12_]^2^⁻in DMF solution upon 320 nm excitation. Kinetic traces and corresponding global fitting curve at **d** 475 nm **e** 600, and **f** 690 nm for [Zr@Sb_12_]^2^⁻in DMF solution upon 400 nm excitation. Kinetic traces and corresponding global fitting curve at **g** 475 nm **h** 600, and **i** 690 nm for [Zr@Sb_13_]^3^⁻in DMF solution upon 320 nm excitation. Kinetic traces and corresponding global fitting curve at **j** 475 nm **k** 600, and **l** 690 nm for [Zr@Sb_13_]^3^⁻in DMF solution upon 400 nm excitation.

**Table S5.** Global lifetime analysis result of [Zr@Sb_12_]^2^⁻ and [Zr@Sb_13_]^3^⁻ in DMF solution upon 320 nm and 400 nm excitation.

|  | *τ*_1_ (ps) | *τ*_2_ (ps) | *τ*_3_ (ps) |
| --- | --- | --- | --- |
| ZrSb_12_ – 320 nm ex. | 2.1 ± 0.5 | 22 ± 7 | 465 ± 91 |
| ZrSb_12_ – 400 nm ex. | 1.7 ± 0.4 | 28 ± 8 | 596 ± 86 |
| ZrSb_13_ – 320 nm ex. | 1.9 ± 0.5 | 18 ± 6 | 106 ± 95 |
| ZrSb_13_ – 400 nm ex. | 1.7 ± 0.5 | 15 ± 6 | 95 ± 91 |

References

(1) Gascoin, F. & Sevov, S. C. Synthesis and characterization of the ‘metallic salts’ A_5_Pn_4_ (A = K, Rb, Cs and Pn = As, Sb, Bi) with isolated zigzag tetramers of Pn_4_^4−^ and an extra delocalized electron. *Inorg. Chem.* **40**, 5177–5181 (2001). https://doi.org/10.1021/ic0104499.
(2) AMS 2024.1, SCM, Theoretical Chemistry, Vrije Universiteit, Amsterdam, The Netherlands, Http://Www.Scm.Com.
(3) van Lenthe, E.; Baerends, E.-J.; Snijders, J. G. Relativistic Total Energy Using Regular Approximations. *J. Chem. Phys.* **1994**, *101* (11), 9783–9792. <https://doi.org/10.1063/1.467943>.
(4) Brémond, E.; Adamo, C. Seeking for Parameter-Free Double-Hybrid Functionals: The PBE0-DH Model. *J. Chem. Phys.* **2011**, *135* (2), 024106. https://doi.org/10.1063/1.3604569.
(5) Versluis, L.; Ziegler, T. The Determination of Molecular Structures by Density Functional Theory. The Evaluation of Analytical Energy Gradients by Numerical Integration. *J. Chem. Phys.* **1988**, *88* (1), 322–328. https://doi.org/10.1063/1.454603.
(6) Klamt, A.; Jonas, V. Treatment of the Outlying Charge in Continuum Solvation Models. *J. Chem. Phys.* **1996**, *105* (22), 9972. https://doi.org/10.1063/1.472829.
(7) Grimme, S. Density Functional Theory with London Dispersion Corrections. *Wiley Interdiscip. Rev. Comput. Mol. Sci.* **2011**, *1* (2), 211–228. https://doi.org/10.1002/wcms.30.
(8) Heine, T.; Corminboeuf, C.; Seifert, G. The Magnetic Shielding Function of Molecules and Pi-Electron Delocalization. *Chem. Rev.* **2005**, *105* (10), 3889–3910. <https://doi.org/10.1021/cr030082k>.
(9) Merino, G.; Heine, T.; Seifert, G. The Induced Magnetic Field in Cyclic Molecules. *Chem. - A Eur. J.* **2004**, *10* (17), 4367–4371. https://doi.org/10.1002/chem.200400457.
(10) Islas, R.; Heine, T.; Merino, G. The Induced Magnetic Field. *Acc. Chem. Res.* **2012**, *45* (2), 215–228. https://doi.org/10.1021/ar200117a.
(11) Autschbach, J. Analyzing NMR Shielding Tensors Calculated with Two-Component Relativistic Methods Using Spin-Free Localized Molecular Orbitals. *J. Chem. Phys.* **2008**, *128* (16). https://doi.org/10.1063/1.2905235.
(12) Krykunov, M.; Ziegler, T.; Lenthe, E. van. Hybrid Density Functional Calculations of Nuclear Magnetic Shieldings Using Slater‐type Orbitals and the Zeroth‐order Regular Approximation. *Int. J. Quantum Chem.* **2009**, *109* (8), 1676–1683. https://doi.org/10.1002/qua.21985.
(13) Baranac-Stojanović, M. New Insight into the Anisotropic Effects in Solution-State NMR Spectroscopy. *RSC Adv.* **2014**, *4* (1), 308–321. https://doi.org/10.1039/C3RA45512B.
(14) Klod, S.; Kleinpeter, E. Ab Initio Calculation of the Anisotropy Effect of Multiple Bonds and the Ring Current Effect of Arenes Application in Conformational and Configurational Analysis. *J. Chem. Soc. Perkin Trans. 2* **2001**, No. 10, 1893–1898. https://doi.org/10.1039/B009809O.
(15) Charistos, N. D.; Papadopoulos, A. G.; Sigalas, M. P. Interpretation of Electron Delocalization in Benzene, Cyclobutadiene, and Borazine Based on Visualization of Individual Molecular Orbital Contributions to the Induced Magnetic Field. *J. Phys. Chem. A* **2014**, *118* (6), 1113–1122. https://doi.org/10.1021/jp411410r.
(16) Schleyer, P. von R.; Maerker, C.; Dransfeld, A.; Jiao, H.; Hommes, N. J. R. van E. Nucleus-Independent Chemical Shifts: A Simple and Efficient Aromaticity Probe. *J. Am. Chem. Soc.* **1996**, *118* (26), 6317–6318. https://doi.org/10.1021/ja960582d.
(17) Zubarev, D. Y.; Boldyrev, A. I. Deciphering Chemical Bonding in Golden Cages. *J. Phys. Chem. A* **2009**, *113* (5), 866–868. https://doi.org/10.1021/jp808103t.
(18) Zubarev, D. Y.; Boldyrev, A. I. Developing Paradigms of Chemical Bonding: Adaptive Natural Density Partitioning. *Phys. Chem. Chem. Phys.* **2008**, *10* (34), 5207–5217. https://doi.org/10.1039/b804083d.
(19) Frisch, M. J.; Trucks, G. W.; Schlegel, H. B.; Scuseria, G. E.; Robb, M. A.; Cheeseman, J. R.; Scalmani, G.; Barone, V.; Mennucci, B.; Petersson, G. A.; Nakatsuji, H.; Caricato, M.; Li, X.; Hratchian, H. P.; Izmaylov, A. F.; Bloino, J.; Zheng, G.; Sonnenberg, J. L.; Had, M.; Fox, D. J.; Hada, M.; Ehara, M.; Toyota, K.; Fukuda, R.; Hasegawa, J.; Ishida, M.; Nakajima, T.; Honda, Y.; Kitao, O.; Nakai, H.; Vreven, T.; Montgomery Jr., J. A.; Peralta, J. E.; Ogliaro, F.; Bearpark, M.; Heyd, J. J.; Brothers, E.; Kudin, K. N.; Staroverov, V. N.; Kobayashi, R.; Normand, J.; Raghavachari, K.; Rendell, A.; Burant, J. C.; Iyengar, S. S.; Tomasi, J.; Cossi, M.; Rega, N.; Millam, J. M.; Klene, M.; Knox, J. E.; Cross, J. B.; Bakken, V.; Adamo, C.; Jaramillo, J.; Gomperts, R.; Stratmann, R. E.; Yazyev, O.; Austin, A. J.; Cammi, R.; Pomelli, C.; Ochterski, J. W.; Martin, R. L.; Morokuma, K.; Zakrzewski, V. G.; Voth, G. A.; Salvador, P.; Dannenberg, J. J.; Dapprich, S.; Daniels, A. D.; Farkas, Ö.; Foresman, J. B.; Ortiz, J. V; Cioslowski, J.; Fox, D. J. Gaussian 09. 2013.
(20) Lu, T. A Comprehensive Electron Wavefunction Analysis Toolbox for Chemists, Multiwfn. *J. Chem. Phys.* **2024**, *161* (8), 082503. https://doi.org/10.1063/5.0216272.
(21) Lu, T.; Chen, F. Multiwfn: A Multifunctional Wavefunction Analyzer. *J. Comput. Chem.* **2012**, *33* (5), 580–592. https://doi.org/10.1002/jcc.22885.
(22) Adamo, C.; Barone, V. Toward Reliable Density Functional Methods without Adjustable Parameters: The PBE0 Model. *J. Chem. Phys.* **1999**, *110*, 6158–6170. <https://doi.org/10.1063/1.478522>.
(23) Weigend, F.; Ahlrichs, R. Balanced Basis Sets of Split Valence, Triple Zeta Valence and Quadruple Zeta Valence Quality for H to Rn: Design and Assessment of Accuracy. *Phys. Chem. Chem. Phys.* **2005**, *7*, 3297–3305. <https://doi.org/10.1039/B508541A>.
(24) VandeVondele, J.; Hutter, J. Gaussian Basis Sets for Accurate Calculations on Molecular Systems in Gas and Condensed Phases. *J. Chem. Phys.* **2007**, *127*, 114105. <https://doi.org/10.1063/1.2770708>.
(25) Koradi, R.; Billeter, M.; Wüthrich, K. MOLMOL: A Program for Display and Analysis of Macromolecular Structures. *J. Mol. Graph.* **1996**, *14*, 51–55. https://doi.org/10.1016/0263-7855(96)00009-4.
(26) Humphrey W, Dalke A, Schulten K. VMD: visual molecular dynamics. *J Mol Graph.* **1996** Feb;14(1):33-8, 27-8. https://doi.org/10.1016/0263-7855(96)00018-5. PMID: 8744570.
(27) Szczepanik, D. W.; Andrzejak, M.; Dominikowska, J.; Pawełek, B.; Krygowski, T. M.; Szatylowicz, H.; Solà, M. The Electron Density of Delocalized Bonds (EDDB) Applied for Quantifying Aromaticity. *Phys. Chem. Chem. Phys*. **2017**, 19 (42), 28970–28981. https://doi.org/10.1039/C7CP06114E.
(28) Szczepanik, D. W. RunEDDB Program. http://aromaticity.eu/eddb/.
(29) Szczepanik, D. W.; Żak, E.; Dyduch, K.; Mrozek, J. Electron Delocalization Index Based on Bond Order Orbitals. *Chem. Phys. Lett*. **2014**, 593, 154–159. https://doi.org/10.1016/j.cplett.2014.01.006.
